# Pipeline for the identification and classification of ion channels in parasitic flatworms

**DOI:** 10.1186/s13071-016-1428-2

**Published:** 2016-03-16

**Authors:** Bahiyah Nor, Neil D. Young, Pasi K. Korhonen, Ross S. Hall, Patrick Tan, Andrew Lonie, Robin B. Gasser

**Affiliations:** Faculty of Veterinary and Agricultural Sciences, The University of Melbourne, Parkville, Victoria 3010 Australia; Genome Institute of Singapore, 60 Biopolis Street, Singapore, 138672 Republic of Singapore; Cancer and Stem Cell Biology, Duke-NUS Graduate Medical School, Singapore, 138672 Republic of Singapore; Victorian Life Sciences Computation Initiative (VLSCI), The University of Melbourne, Parkville, Victoria 3010 Australia

**Keywords:** Ion channels, Identification, Classification, Parasitic flatworms, Bioinformatic pipeline

## Abstract

**Background:**

Ion channels are well characterised in model organisms, principally because of the availability of functional genomic tools and datasets for these species. This contrasts the situation, for example, for parasites of humans and animals, whose genomic and biological uniqueness means that many genes and their products cannot be annotated. As ion channels are recognised as important drug targets in mammals, the accurate identification and classification of parasite channels could provide major prospects for defining unique targets for designing novel and specific anti-parasite therapies. Here, we established a reliable bioinformatic pipeline for the identification and classification of ion channels encoded in the genome of the cancer-causing liver fluke *Opisthorchis viverrini*, and extended its application to related flatworms affecting humans.

**Methods:**

We built an ion channel identification + classification pipeline (called MuSICC), employing an optimised support vector machine (SVM) model and using the Kyoto Encyclopaedia of Genes and Genomes (KEGG) classification system. Ion channel proteins were first identified and grouped according to amino acid sequence similarity to classified ion channels and the presence and number of ion channel-like conserved and transmembrane domains. Predicted ion channels were then classified to sub-family using a SVM model, trained using ion channel features.

**Results:**

Following an evaluation of this pipeline (MuSICC), which demonstrated a classification sensitivity of 95.2 % and accuracy of 70.5 % for known ion channels, we applied it to effectively identify and classify ion channels in selected parasitic flatworms.

**Conclusions:**

MuSICC provides a practical and effective tool for the identification and classification of ion channels of parasitic flatworms, and should be applicable to a broad range of organisms that are evolutionarily distant from taxa whose ion channels are functionally characterised.

**Electronic supplementary material:**

The online version of this article (doi:10.1186/s13071-016-1428-2) contains supplementary material, which is available to authorized users.

## Background

Ion channels are pore-forming transmembrane protein complexes, whose functions include generating electrical signals (action potentials) by regulating the flow of ions across the membranes of cells, gating ion flow across epithelial and secretory cells, and governing cell volume [1]. These channels are categorised physiologically based on their gating mechanisms (voltage-gated or ligand-gated) and the types of ions that they transport (e.g., Ca^2+^, Cl^−^, K^+^ and Na^+^) [1, 2]. Given that they have essential and specific roles in a wide range of different cells and that the disruption or mutation of their functions often causes serious disease [3], ion channels are recognised as valuable targets for drugs for many non-infectious disorders of humans and animals [4, 5].

Ion channel repertoires of some (“model”) organisms, such as *Homo sapiens* (human) and *Caenorhabditis elegans* (free-living roundworm), are relatively well defined, because of the availability of extensive genomic, proteomic, functional and other datasets as well as ion channel functional information for these species (e.g., [6–10]), but this is not the case for most other organisms whose biology, biochemistry and physiology are largely unknown and are divergent from well-characterised organisms, such as humans and *C. elegans* [11]. This is particularly the case for eukaryotic pathogens, such as flatworm parasites (phylum Platyhelminthes), which are evolutionarily distinct from “model” species and cause devastating diseases of major proportion in humans and animals around the world [12].

As the management of many socioeconomically important parasitic flatworm diseases is often inadequate or compromised due to the inefficacy of some anthelmintics or emerging resistance [13, 14], identifying and characterising ion channel repertoires in flatworms could define novel and selective drug targets, and might open up avenues to design safe drugs with essentially no adverse effect on the human or animal hosts. Clearly, the massive expansion of genomic and transcriptomic datasets for a range of important parasitic flatworms, such as *Opisthorchis viverrini, Clonorchis sinensis* (liver flukes), *Schistosoma haematobium*, *Schistosoma japonicum*, *Schistosoma mansoni* (blood flukes), *Echinococcus granulosus*, *Echinococcus multilocularis* and *Taenia solium* (see Table 1), provides enormous scope to investigate the repertoires of ion channels in such worms. However, given their substantial molecular genetic and evolutionary divergence from well-characterised organisms, the challenge now is to reliably predict or identify as well as classify these channels from available molecular datasets.

**Table 1:** Salient information on parasites chosen for the present study

Class/Family	Species	Disease	Key references
Trematoda			
Opisthorchiidae (liver fluke)	*Opisthorchis viverrini*	Opisthorchiasis; cholangiocarcinoma	[27, 34]
	*Clonorchis sinensis*	Clonorchiasis; cholangiocarcinoma	[62, 63]
Schistosomatidae (blood fluke)	*Schistosoma haematobium*	Urogenital schistosomiasis; squamous cell carcinoma of the bladder	[64, 65]
	*Schistosoma japonicum*	Hepatointestinal schistosomiasis	[66, 67]
	*Schistosoma mansoni*	Hepatointestinal schistosomiasis	[68–70]
Cestoda			
(Taeniidae)	*Echinococcus granulosus*	Cystic echinococcosis or hydatidosis	[28, 71]
	*Echinococcus multilocularis*	Alveolar echinococcosis or hydatidosis	[28, 71]
	*Taenia solium*	Cysticercosis	[29, 71]

The availability of large genomic datasets and the development of new bioinformatic approaches now make it feasible to classify ion channels using amino acid sequence and/or protein structural similarities. Generic bioinformatic tools, such as BLAST [15], HMMER [16] and InterProScan [17], are commonly used for gene annotation [18–20]. Besides these generic tools, some studies [21–23] have delivered algorithms specifically to classify ion channels, and most of them employ machine-learning algorithms trained using ion channel protein sequence data from specialised protein databases, such as IUPHAR (International Union of Basic and Clinical Pharmacology), LIC (ligand-gated ion channel) and VKCDB (voltage-gated potassium channel) [24–26]. Most functionally annotated and curated ion channels in the UniProtKB/SwissProt database are from deuterostomes (e.g., vertebrates) and ecdysozoans (e.g. *C. elegans* and *Drosophila melanogaster*). The integration of these data and use of advanced bioinformatics should significantly enhance our ability to explore (identify and classify) ion channels in eukaryotes that are evolutionarily distant from taxa whose ion channels are functionally characterised. To this end, the aim of this study was to establish a bioinformatic pipelines for the reliable identification and classification of ion channels in parasitic flatworms affecting millions of people and animals worldwide (Table 1). Our main focus here was on the cancer-causing (carcinogenic) liver fluke *O. viverrini* [27], and we extended its application to related flukes as well as socioeconomically important tapeworm parasites [28, 29] (Table 1).

## Methods

**Fig. 1:**
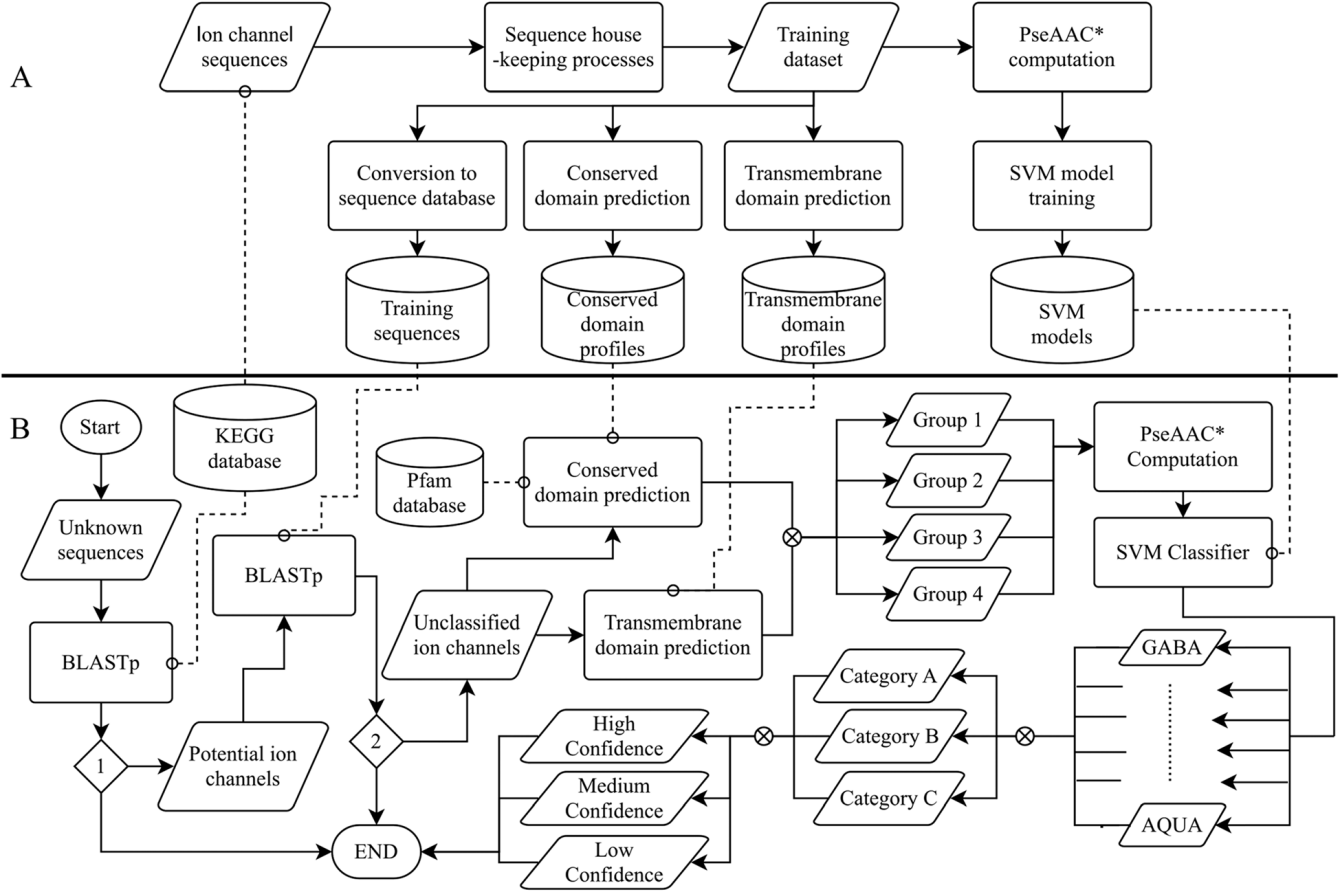
Development (training processes) and implementation of the bioinformatics pipeline, designated multi-screening ion channel classifier (MuSICC). Panel A shows the training processes, starting from the selection of the training dataset to the construction of databases for subsequent implementation. Panel B shows the workflow to predict and classify unknown sequences. Both diamonds (1 and 2) “decide” whether a sequence shares significant similarity to known ion channel sequences, employing the KEGG and training sequences databases. Asterisk denotes pseudoamino acid composition (PseAAC) computation

We constructed and assessed a bioinformatics pipeline, called multi-screening ion channel classifier (MuSICC), to identify and classify ion channels (Fig. 1). This pipeline, which uses three existing bioinformatic tools and a support vector machine (SVM), was trained using known ion channel sequences obtained from public databases. Known sequences were subjected to multiple screening processes before being used to build the SVM models. For training, four internal databases were constructed: (i) known ion channel sequences used to construct the pipeline; (ii) conserved domain profiles for ion channels, (iii) transmembrane domain profiles for ion channels, and (iv) SVM models. The datasets as well as methods used for the prediction of ion channel proteins, and the construction and testing of SVMs are described in the following:

### Datasets

Three datasets were prepared: (1) The *training dataset* was established using all classified ion channel and aquaporin sequence data from the KEGG database [30, 31] as well as molluscan ion channel sequences in the UniProtKB/Swiss-Prot Database [32], sodium channel protein 1 brain (Q05973; SCN1_HETBL), glutamate receptor (P26591; GLRK_LYMST), gamma-aminobutyric acid receptor subunit beta (P26714; GBRB_LYMST) and FMRFamide-activated amiloride-sensitive sodium channel (Q25011; FANA_HELAS). All UniProt/Swiss-Prot sequences were annotated using the KEGG orthology ion channel K-term of the KEGG entry, with highest sequence similarity inferred using BLASTp [15]. All human and *C. elegans* sequences and any sequences with ambiguous amino acid residues (“X”, “B” or “Z”), or that were annotated as “hypothetical” or “putative”, were removed from the training dataset. The training dataset was divided into 48 ion channel subfamily classes and one aquaporin class. Sequence similarity bias was removed from each subfamily class by selecting representative protein sequences of particular groups with >80 % sequence similarity using the CD-HIT program [33]. (2) The *test dataset* was established using all predicted proteins available for proteomes of human and *C. elegans* in the KEGG database [30, 31]. (3) The *parasite dataset* represented amino acid sequences translated from genes of *O. viverrini* [34] and related flatworms *Cl. sinensis* (liver flukes), *S. haematobium*, *S. japonicum*, *S. mansoni* (blood flukes), *E. granulosus*, *E. multilocularis* and *T. solium* (tapeworms) (Table 1).

### Prediction of ion channel proteins

For the *test* and *parasite datasets*, ion channels were predicted based on amino acid sequence similarity searches (Fig. 1). To remove any ‘false-positives’ from these datasets, we initially screened each sequence against the KEGG database using BLASTp [15] (E-value of <10^−15^), retaining proteins with a best match to an annotated ion channel. For the *test dataset*, a sequence similarity match to a human or *C. elegans* sequence in the KEGG database was ignored. Then, the remaining *test* and *parasite dataset* proteins were compared (BLASTp, E-value <10^−45^) against the *training dataset*, with sequences similar to *training dataset* proteins retained as putative ion channel proteins.

For all sequences in each dataset, we identified conserved domains using InterProScan v.5.7.48 [35] and the Pfam database [36]. We curated the Pfam conserved domain accession numbers for individual sequences in the *training dataset*, to create conserved (C-) domain profiles for individual ion channel subfamilies. These profiles were then used to characterise and group sequences in the *test* and *parasite ion channel datasets*, based on the presence or absence of C-domains. Then, we predicted transmembrane (TM-) domains in individual sequences using TMHMM v.2.0 [37] and curated the number of TM-domains predicted from each sequence in the *training dataset* for each ion channel subfamily. The range of predicted TM-domains for sequences classified in each subfamily was then used to characterise and group sequences in *test* and *parasite ion channel datasets*. Finally, we divided putative *test* and *parasite* ion channels into four distinct groups according to: sequence similarity to known ion channels, and presence of C-domain and TM-domain(s) (*Group 1*); similarity, and presence of C-domain, but no TM-domain(s) (*Group 2*); similarity, and presence of TM-domain(s), but no C-domain (*Group 3*); similarity, but no C- or TM-domains (*Group 4*).

### Construction and testing of support vector machines (SVMs)

For each sequence in each dataset, we constructed the pseudo-amino acid composition [38] with *λ* = 55, weight = 0.7 and using established hydrophobicity values [39], hydrophilicity values [40] and side chain mass values [41]. We also determined the 400 character, dipeptide composition of each sequence in the dataset. The dipeptide composition [*f*(*x*,*y*)] of any combination of two amino acid residues represented as *x* and *y*, for each sequence was computed as

$$ sign\left({a}_i,{a}_{i+1}\right)=\left\{\begin{array}{c}\hfill 0\  if\ {a}_i,{a}_{i+1}\ne xy\hfill \\ {}\hfill 1\  if\ {a}_i,{a}_{i+1}=xy\hfill \end{array}\right. $$

$$ f\left(x,y\right) = \frac{{\displaystyle {\sum}_{i=1}^{n-1}} sign\left({a}_i,{a}_{i+1}\right)}{n-1} $$

where *n* is the length of the sequence and *a*_*i*_ represents amino acid residue at position *i*. In total, each sequence was represented as a vector of 475 features, including the amino acid composition (20 characters), Chou’s pseudo-amino acid composition (*λ* = 55) and dipeptide frequency (400 characters).

The SVMs were constructed using LIBSVM [42] extension in R v.3.2.0 [43] using the *e1071* package [44]. For comparative purposes, five models were constructed using radial basis kernel, each with different sets of features and kernel parameters that were tuned with five-fold cross validation. The first model, named ‘Amino’, was built using 20 amino acid frequencies as features; the second model, called ‘Chemistry’, was built using 55 features based on the hydrophobicity, hydrophilicity and side chain-mass. The third model, ‘Chou’, was built using Chou’s pseudo-amino acid composition by combining the 20 amino acid and 55 chemical information features. The fourth model, named ‘Dipeptide’, was built using 400 dipeptide composition features. The last model, ‘Classifier’, was built using all 475 features.

The classification models were validated using five-fold cross-validation, and assessed against the classifications of known ion channel and aquaporin sequences encoded in the human and *C. elegans* genomes. Receiver operating characteristic (ROC) analysis [45] was conducted to evaluate the performance of each model. For comparative purposes, we also assessed the *test dataset* using other probabilistic classification methods, including random forest, classification *via* logistic regression and prior classifier, conducted using established methods [46–48]. Using the best-performing classification models, confusion matrices were constructed to further evaluate each model and compare their performance based on the final table of confusion. For the final model, the average classification probability values for individual subfamilies in the *test dataset* were computed; these probability values were utilised to classify the ion channels predicted from the *parasite dataset.*

Protein categories were classified based on SVM probability values: *Category A* proteins had probability values greater than or equal to the subfamily probability threshold. *Category B* proteins had probability values between 50 % of the subfamily probability threshold and the subfamily probability threshold. *Category C* proteins had probability values less than 50 % of the subfamily probability threshold. A confidence ranking was given to our ion channel classifications. *High confidence* classifications included channels in *Category A* (*Groups 1* to *4*) and *Category B* (*Groups 1* and *2*), which were annotated by SVM subfamily classification. *Medium confidence* classifications included channels in *Category B* (*Groups 3* and *4*), which were annotated by SVM subfamily-classifications and designated with the suffix, “-like” (e.g. GABA-like ion channel). *Low confidence* classifications included all proteins in *Category C* (*Groups 1* to *4*), which represented ion channel-like proteins but could not be confidently assigned to a particular family or subfamily.

## Results

### Training and test datasets

The *training dataset* consisted of 26,050 classified ion channel and aquaporin sequences (Additional file 1: Table S1). After removing protein sequences from human and *C. elegans* as well as ambiguous sequences and sequence similarity bias from the dataset, 6299 classified ion channel and aquaporin sequences remained for model construction and training (Additional file 1: Tables S1 and S2). The *test dataset* consisted of the combined human and *C. elegans* proteins, including 389 sequences annotated with ion channel and aquaporin K-terms in the KEGG database (Additional file 1: Table S1).

### Identification of ion channels

From the *test dataset*, 657 ion channel-like proteins with sequence similarity (BLASTp, E-value <10^−15^) to known ion channels in the KEGG database were identified (Additional file 1: Table S3); they included 390 and 267 from humans and *C. elegans*, respectively, of which 299 human (100 %) and 93 *C. elegans* (100 %) ion channels were retained. Using a stringent sequence similarity search (BLASTp, E-value <10^−45^) against sequences in the training dataset, 344 human and 185 *C. elegans* sequences were retained (Additional file 1: Table S3), including 299 human (100 %) and 93 *C. elegans* (100 %) ion channels.

A total of 194 unique Pfam C-domains were detected in 6161 sequences (~97.8 %) of the training dataset, with 88 unique C-domains detected in >75 % of the sequences of 45 ion channel subfamilies (Additional file 2: Figure S1), such as the neurotransmitter-gated ion channel ligand-binding domain (PF02931) in >88 % of the Cys-loop subfamilies. TM-domains were detected in 5774 (~91.7 %) sequences in the training set, with the number of such domains varying from 1 to 22 per protein (Additional file 2: Figure S2), being within the expected range for individual ion channel subfamilies. TMs were not detected in 525 sequences (Additional file 2: Figure S2). Based on sequence similarity, and the presence/absence of conserved and TM-domains, the sequences from the *test dataset* were divided into *Group 1* (*n* = 443; including 335 known ion channels), *Group 2* (57; 44 known ion channels), *Group 3* (15; 5 known ion channels) and *Group 4* (14; 5 known ion channels). Sequences within individual groups were then subjected to ion channel classification (Additional file 1: Table S4).

### Ion channel classifiers

The performance of each of the five SVM models to classify ion channels was assessed using the *training dataset*. For this purpose, any known non-ion channel sequences were removed. Based on the five-fold cross-validation, training and test accuracies (Additional file 1: Table S5), we concluded that the ‘Dipeptide’ (94.6 % test accuracy) and ‘Classifier’ (95.9 % test accuracy) models out-performed the other three models (Additional file 1: Table S5). Confusion matrices for the ‘Classifier’ and ‘Dipeptide’ models were constructed to further evaluate the models, and to compare their performances based on the final table of confusion (Additional file 1: Table S6); the ‘Classifier’ model recorded the best overall scores (Additional file 1: Table S6).

The performance of the ‘Classifier’ model was evaluated using the complete *test dataset* (including protein sequences that were not ion-channels) and recorded a sensitivity of 95.2 %, an accuracy of 70.5 % and a specificity of 0 %; this result was expected, as an SVM model had not been trained for protein sequences other than ion channels (i.e. “non-ion channel” sequences). This finding shows the importance of identifying ion channels prior to classifying them.

The performance of the SVM classifier and the other probabilistic classification methods (random forest, classification *via* logistic regression and prior classifier) were then compared using the *test dataset*, employing the sorted probability values to construct ROC curves for each classifier (Additional file 2: Figure S3). The area-under-the-curve (AUC) for the SVM ‘Classifier’ was 0.911, random forest classification was 0.9105, the logistic regression classifier was 0.8211 and the AUC for prior classifier was 0.6701. The SVM and random forest classifiers performed similarly, but due to the high dimensionality of the data, classification *via* SVM was preferred.

Overall, there was a correlation between the probability values for the *test dataset* and correctness of their classification (Additional file 2: Figure S4A). In general, classifications with probability values of ≥0.54 tended to be correct, whereas those with lower probability values tended to be incorrect. When probability values were compared among ion channel subfamilies (Additional file 2: Figure S4B), the average probability values for each subfamily ranged from ~ 0.15 to 0.91 (Additional file 2: Figure S4B). Based on these findings, we elected to infer confidence in future classifications made using the SVM classifier employing the average probability values for individual subfamilies (Additional file 2: Figure S4B), instead of using single threshold probability value for all ion channel classifications (Additional file 2: Figure S4A). Using the *test dataset*, we observed higher probability values for proteins identified as *Group 1* and *Group 2* ion channels (Additional file 2: Figure S5). The majority of ion channels in *Groups 3* and *4* had classifier probability values of <0.5 (Additional file 2: Figure S5).

### Ion channels of *Opisthorchis viverrini* and other flatworms

**Fig. 2:**
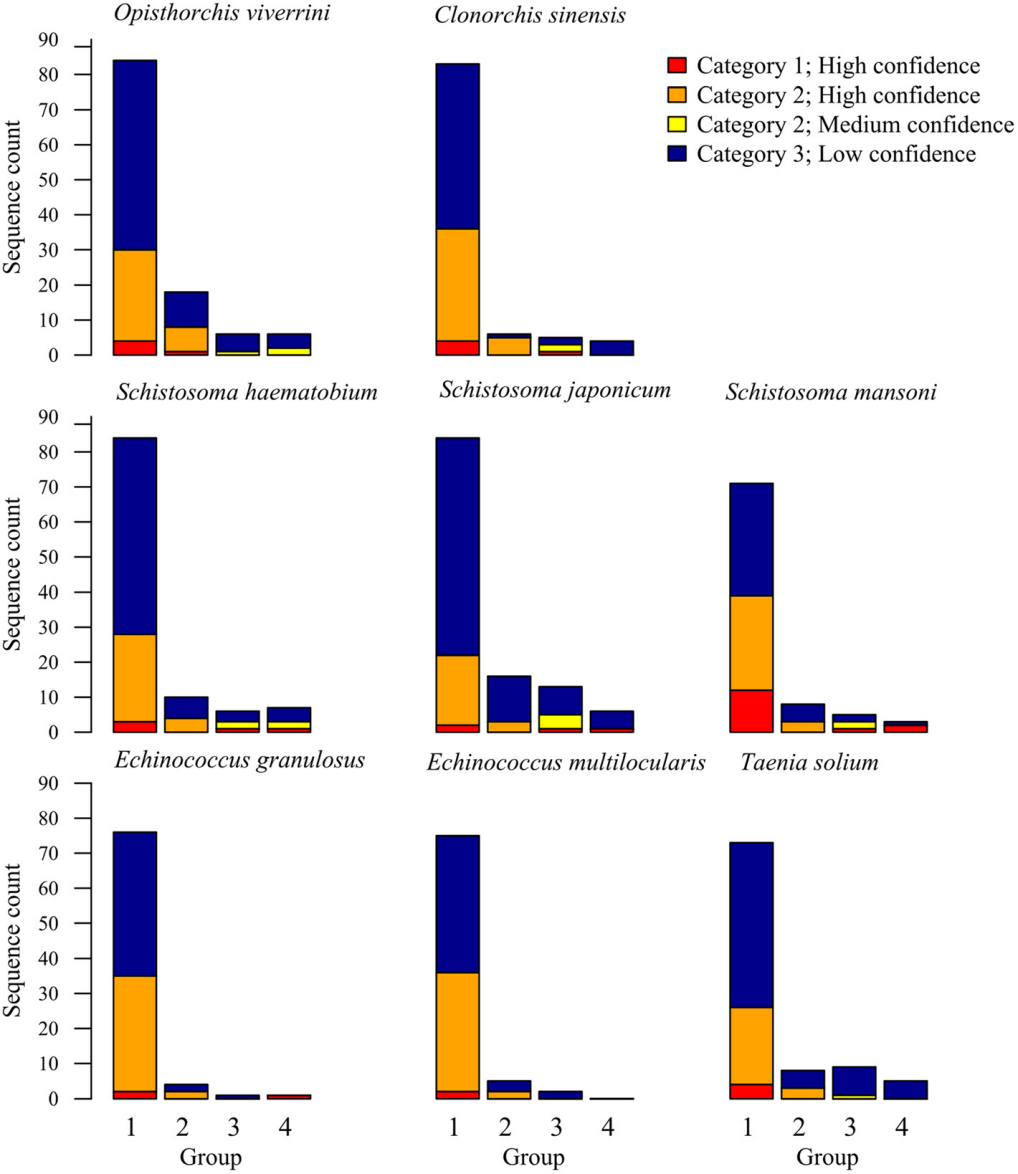
Confidence in ion channel classifications for selected parasitic flatworms by group and classification category

Using our MuSICC pipeline (Fig. 1b; available for download at https://github.com/vetscience/ion-channel-classifier), a total of 114 ion channels were predicted (in 53 h using an Intel ES-2695 2.4 GHz processor with eight cores) from the draft genome of *O. viverrini* by BLASTp against the KEGG database and the training dataset, and identified the presence/absence of C- and TM-domains. Thereafter, these sequences were divided (in 3 min) into the following groups: 84 sequences shared sequence similarity to known ion channels and contained ion channel C- and TM-domains (*Group 1*); 18 sequences with sequence similarity to an ion channel and expected C-domains, but lacked the expected TM-domain profile (*Group 2*); six sequences were similar to known ion channels contained an expected TM-domain profile but lacked the expected C-domains (*Group 3*) and six sequences shared similarity to a known ion channel but did not contain expected C- and TM-domains (*Group 4*) (Fig. 2 and Additional file 1: Table S7).

These 114 predicted ion channels were classified using the established SVM classifier and average probability value thresholds for individual ion channel subfamilies (Fig. 2 and Table 2), 38 of which were classified, with high confidence, and three as sub-family-like ion channels proteins with medium confidence; and 73 were classified as ion channel-like proteins (with no family or sub-family assignment); 30 of 38 sequences classified with high confidence were grouped in *Group 1*, and eight were in *Group 2*. One of the sequences classified with medium confidence was in *Group 3* and the other two were in *Group 4*. Of the 73 sequences classified with low confidence, 54 were in *Group 1*, 10 in *Group 2*, five in *Group 3* and four in *Group 4* (Fig. 2). Then, the classification of ion channel sequences of *O. viverrini* was compared with those from human and *C. elegans* (Table 2). There were notable differences in the numbers of sequences for individual ion channel families between protostomes (*O. viverrini* and *C. elegans*). For *O. viverrini*, we classified eight calcium ion channels; five voltage-gated calcium ion channels; a ryanodine receptor, three CatSper, and two-pore channels. Therefore, *O. viverrini* was shown to have slightly more calcium ion channels (*n* = 8) compared with *C. elegans* (*n* = 6). The classification of *O. viverrini* ion channels showed a considerably higher number of sequences (*n* = 3) representing the “Epithelial and Related Channels” ion channel family compared with *C. elegans* (*n* = 0). One of “Epithelial and Related Channels” family in *O. viverrini* sequences was classified as a acid-sensing ion channel (ASIC), and two sequences as ATP-gated cation channels (P2X).

**Table 2:** Comparison of the numbers of ion channel sequences within each family among humans, *C. elegans* and representative parasitic flatworms^*^ that were classified with high and medium confidence

Ion channel family	*Hs*	*Ce*	Trematoda					Cestoda		
			*Ov*	*Cs*	*Sh*	*Sj*	*Sm*	*Eg*	*Em*	*Ts*
Cys-loop superfamily	46	36	3	2	4	5	4	6	6	7
Glutamate-gated cation channels	18	8	4	6	6	0	4	5	5	5
Epithelial and related channels	16	0	3	2	1	3	4	2	2	3
Ryanodine and IP3 receptors	6	2	1	1	1	1	2	1	1	0
Voltage-gated ion channels	114	22	16	15	14	11	16	12	13	7
Related to voltage-gated ion channels	66	14	10	13	9	6	12	9	8	7
Chloride channels	19	7	2	2	2	3	3	3	3	1
Aquaporins	14	4	2	3	1	2	2	0	0	0
Unclassified ion channel-like proteins	-	-	73	54	69	88	40	38	44	65
Total	299	93	114	98	107	119	87	82	82	95

*Hs *Homo* *sapiens*, *Ce* *Caenorhabditis elegans*, *Ov* *Opisthorchis viverrini*, *Cs* *Clonorchis sinensis*, *Sh* *Schistosoma haematobium*, *Sj* *Schistosoma japonicum*, *Sm* *Schistosoma mansoni*, *Eg* *Echinococcus granulosus*, *Em* *Echinococcus multilocularis*, *Ts* *Taenia solium*

**Fig. 3:**
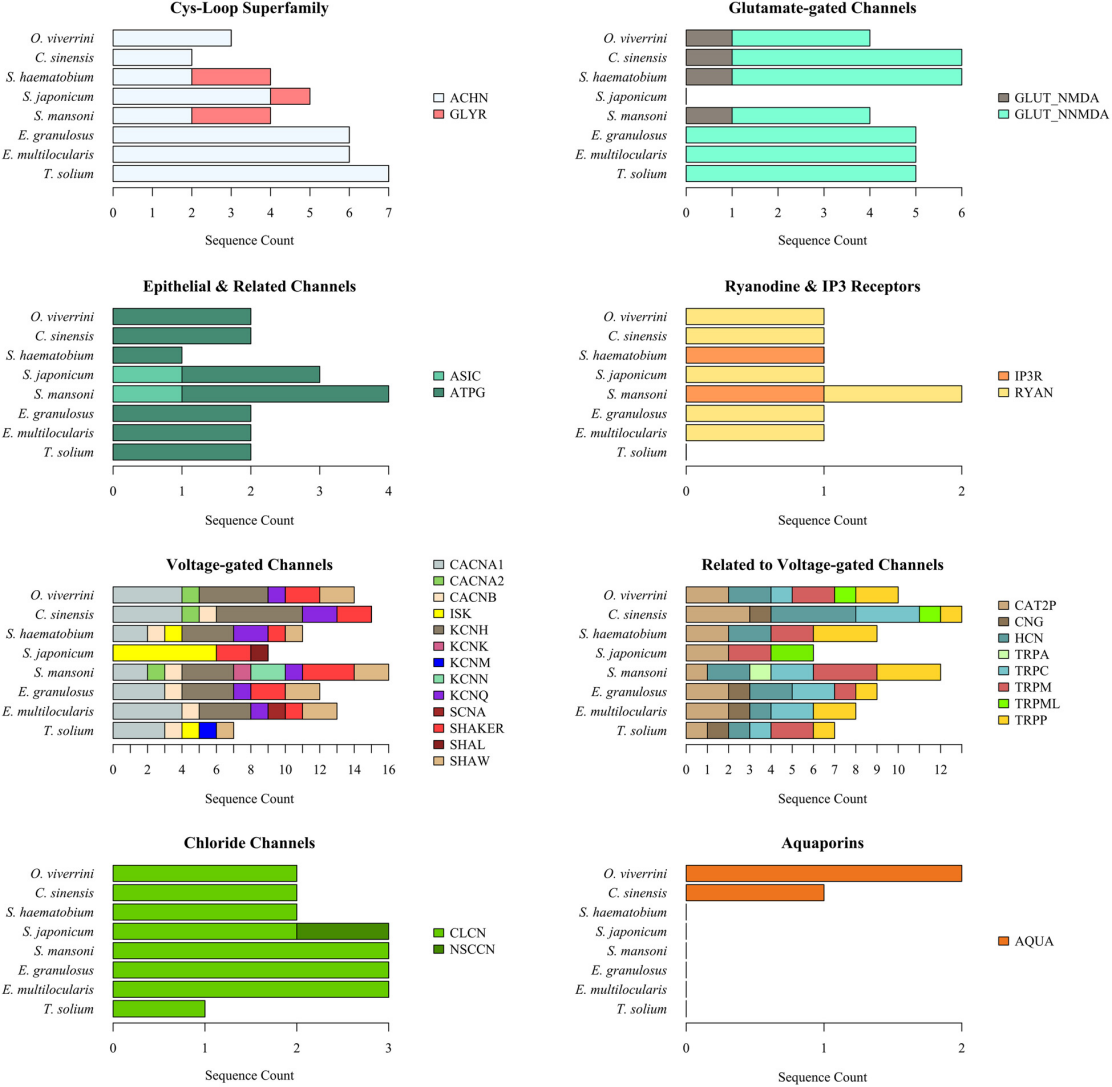
Summary of flatworm proteins classified to ion channel family and subfamily with high confidence. Ion channel subfamily abbreviations are described in Additional file 1: Table S7

Logically extending this work, ion channel sequences from other flatworms, including *Cl. sinensis* (liver fluke), *S. haematobium*, *S. japonicum*, *S. mansoni* (blood flukes), *E. granulosus*, *E. multilocularis* and *T. solium* (tapeworms), were predicted and classified using the established pipeline (Figs. 2 and 3, Table 2 and Additional file 1: Tables S7 and S8). For liver flukes, there were no marked differences in the number of sequences classified in individual ion channel families between *O. viverrini* and *C. sinensis*, although more voltage-gated cation channels could be classified with high confidence for the former species (*n* = 16) than the latter (*n* = 15) (Fig. 3 and Additional file 1: Table S8). For blood flukes, the total number of classified ion channel sequences varied considerably among *S. haematobium*, *S. japonicum* and *S. mansoni* (Figs. 2 and 3, Table 2 and Additional file 1: Tables S7 and S8). The most notable difference was in the number of sequences classified as Isk potassium ion channel (K^+^ channel, KCNE, [Isk]), with *S. japonicum* and *S. haematobium* having eight and four, respectively and *S. mansoni* having none. Although glycine receptors were not classified for liver flukes, *S. haematobium* and *S. mansoni* both had two, *S. japonicum* had one. For tapeworms, there were three differences among the three species; (i) *E. granulosus* and *E. multilocularis* had three Ether-a-go-go potassium channels each, and *T. solium* had none. (ii) *T. solium* one ASIC, the other species had none. (iii) *E. granulosus* and *E. multilocularis* each had one Ryanodine receptor, and *T. solium* had none. Most ion channels were relatively conserved among trematodes, even though the numbers of genes classified in individual families varied. For instance, we predicted and classified the P2X receptor (not conserved with *C. elegans*) in all eight species. In contrast, comparisons showed that the glycine receptors were not conserved between trematodes and cestodes. While glycine receptor genes could be classified for blood flukes, none were classified for the other flatworms studied. We also noted that there are more CatSper and two-pore channel genes classified in trematodes (average of three per species) than in the tapeworms (one per species) (Table 2 and Additional file 1: Tables S7 and S8).

## Discussion

Here, we constructed a practical bioinformatic pipeline, designated MuSICC, to both identify and classify known ion channel families/subfamilies by combining three existing tools and an SVM classifier [49] trained using classified ion channel amino acid compositions, Chou’s pseudo-amino acid compositions [38] and dipeptide frequencies. Although previous tools were developed to identify select ion channel groups [21, 23, 50, 51], none of them both identify and classify (all) ion channels into families and subfamilies. Here, we focused on developing a pipeline that would identify and classify such ion channels from eukaryotic organisms that are genetically and biologically very distinct from “model” organisms (such as *C. elegans*, *Drosophila* and humans, whose ion channels are well-characterised). The phylogenetic positions of parasitic flatworms in the eukaryotic evolutionary tree [52] made them ideal candidates for this study. Moreover, evidence that some flatworms are developing resistance against some of the recommended chemotherapies [13, 14] necessitates the search for new anthelmintics, and ion channels represent promising targets for such drugs [4, 5].

In this study, we first constructed and evaluated the pipeline to identify and classify channels in *O. viverrini*, a highly significant carcinogenic parasite affecting >8 million people worldwide [27]. Following this evaluation, we then applied this pipeline to datasets for seven other socioeconomically important flatworms (Table 1), and undertook a detailed, comparative analysis. The key to accurate identification and classification was the prediction process. As the SVM models were not trained using non-ion channel sequences (i.e. there is non-ion channel classifier), these models are not able to distinguish between ion channel and non-ion channel sequences. Therefore, it is important that the prediction of ion channel sequences (data screening) is accurate. We defined three prediction criteria: (1) significant sequence similarity to known ion channels, (2) presence of ion channel C-domains, and (3) an appropriate number of TM-domains compared with known ion channels.

The sequence similarity (BLASTp) screening steps proved to be effective in filtering out the majority of non-ion channel sequences. In the *test dataset*, 137 sequences (25.9 %) were incorrectly identified as ion channels. We determined that 32 of the 137 ‘false-positives’ did not encode ion channels but were very similar to the ion channel training sequences, whereas 105 sequences were not annotated using the KEGG database. We compared the annotations of these 105 sequences with those in the UniProtKB [53] and RefSeq [54] databases; 88 of the sequences were putative ion channels/proteins, and 17 were unknown/uncharacterised proteins. Therefore, we are confident that future predictions, based on the thresholds set here, will yield a low number of false-positive results, if any at all.

Although conducting two BLASTp processes may be computationally exhaustive and somewhat time consuming, the same result was not achievable by conducting BLASTp only once against either KEGG database or the training sequences. Proteins that were not ion channels and shared high sequence similarity (BLASTp, E-value < 10^−45^) with ion channels were first identified and excluded by initially screening against the complete KEGG database and selecting proteins with a match to an ion channel. An additional search of our curated *training dataset* ensured that false-positive results were minimised, and known ion channels were retained. As the accurate prediction of ion channels is the key to the performance of the present pipeline, we considered the computation time to be less of a priority, at this stage.

The application of three existing bioinformatics tools posed some limitations on the present pipeline. First, the pipeline is dependent on the KEGG database and the KEGG Orthology (KO) grouping method. KO grouping provided a hierarchical annotation based on K-terms, which eased the process of predicting ion channel sequences following the first BLASTp step. However, the implementation of the KO grouping method for predicting sequences was restricted to the annotated ion channel genes in the KEGG database. BLASTp analysis against protein databases without an established annotation system would make an automation process impossible, because manual annotation of ion channel sequences is not feasible as the number of sequences increases. An alternative to the KO annotation is the UniProt Gene Ontology Annotation (UniProt-GOA) database [55]. Second, the bioinformatic pipeline is dependent on the performance of the prediction tools applied – BLASTp, InterProScan and TMHMM 2.0. Based on the present findings, the tools applied here allow the reliable prediction of ion channel sequences. However, the quality of sequences to be identified and classified needs to be high; the use of poor quality sequences will result in mis-classifications.

Two factors were considered crucial in relation to accepting or rejecting the classification made by the SVM classifier. The first was the probability value, computed by the classifier to determine the probability that an unknown sequence belonged to the classified ion channel subfamily, and enabling the probability thresholds to be defined for individual subfamilies (Additional file 2: Figure S4). The second factor considered was the groupings that were made based on the prediction criteria. There was a close association between grouping and the SVM classifier probability value (Additional file 2: Figure S5A); sequences classified with a probability of >0.8 were usually assigned to *Groups 1* and *2* -  the sequences with significant similarity to known ion channels and contained conserved domains of ion channels. Therefore, sequence grouping also provided confidence in the classification of ion channels.

Ion channels are of critical importance for the growth and development of flatworms [56] as well as neuromuscular function [57, 58]. Ion channels can also play an important role in antiparasitic drug activity. For instance, calcium channels are thought to regulate praziquantel’s disruption of Ca^2+^ homeostasis in adult worms [59] and nicotinic acetylcholine receptors (nAChRs) are targets of commercially available drugs that kill nematodes [60]. Most functionally characterised flatworm channels (Table 3) were identified and/or correctly classified using our bioinformatic pipeline (see Additional file 1: Table S8). Only four functionally characterised *S. mansoni* glutamate-gated chloride channel (SmGluCl) subunits [61] appeared to be misclassified using our pipeline, two (*Smp_096480* and *Smp_015630*) of which were classified as glycine receptors in *Category A/Group 1*. In this instance, the accuracy of our classification is likely affected by a lack of conserved features or amino acid sequence between this novel flatworm clade of SmGluCl-like channels and functionally similar receptors in other eukaryotes [61]. Therefore, the under-representation of taxon-specific protein families in public sequence databases can affect the accuracy of the protein classifiers constructed using amino acid features; this observation emphasises the importance of a continual deposition of sequence data for non-model species into public databases. Furthermore, it is expected that improved draft genomes for parasitic flatworms are likely to enhance the predictions of ion channels and other genes. Despite the limitations of current ‘omic resources for non-model species, our pipeline successfully classified a large proportion of the flatworm channels, many with high confidence. These data were used to explore similarities and differences in ion channel subfamilies between flatworms and model organisms.

**Table 3:** Ion channels of parasitic flatworms described in the published literature and whether they were identified and classified correctly using our bioinformatic pipeline (MuSICC)

Name	Protein ID	Species	Identified/characterised^*^	Classified	Key references
Shaker-related K^+^ channel	SKv1.1	*Schistosoma mansoni*	1/1	1	[72]
P2X receptor	*Sch*P2X	*Schistosoma mansoni*	3/3	3	[73]
Nicotinic acetylcholine receptors	*Sh*AR2beta	*Schistosoma haematobium*	2/1	2	[60]
Ca^2+^ channel beta subunits	-	*Schistosoma mansoni*	1/1	1	[74, 75]
Novel glutamate-gated chloride channel subunits	*Sm*GluCl	*Schistosoma mansoni*	4/4	0	[61]
Acetylcholine-gated chloride channels	*Sm*ACCs	*Schistosoma mansoni*	3/2	0	[19]
ATP-sensitive potassium channel	*Cs*Kir6.2	*Clonorchis sinensis*	0/1	0	[76]
Aquaporins	*Ov*AQP	*Opisthorchis viverrini*	2/1	2	[77, 78]

^*^ Number of ion channels submitted to Swissprot (http://www.uniprot.org/) that are associated with the key references

The number of calcium ion channels classified for *O. viverrini* was higher than for *C. elegans* and humans. The number of sequences encoding such channels in *O. viverrini* represents ~ 19.5 % of 41 sequences classified with confidence to encode ion channels. This is more than the proportion of calcium ion channels in *H. sapiens* (~13.7 %), and there was also considerable diversity compared with *C. elegans* and human. Although there are some channels (~40.8 %) that are conserved among the three species, there are ion channels that are shared only by any two of these organisms. Notably, the acid-sensing ion channels (ASIC) and ATP-gated cation channels (P2X) present in both *O. viverrini* and human were absent from *C. elegans*.

The subsequent classification of ion channels from the seven other species of flatworms (trematodes and cestodes) further reinforced the genetic diversity between these parasites and the two well-characterised “model” organisms. The average probability values, which were lower than the thresholds computed by the SVM classifier, indicated that ion channels of these parasites are distinct from all presently known ion channels, despite being similar to them and containing the C-domains. Furthermore, more than half of the sequences were annotated as “unclassified ion channel-like proteins” based on the low probability values and the absence of ion channel C-domains. Importantly, the bioinformatics pipeline established here is able to identify and classify ion channels (with 95 % accuracy), irrespective of sequence diversity. Nonetheless, it may be possible, in the future, to enhance the performance of the pipeline using structural similarity predictions and by training the SVM classifier using protein sequences other than ion channel to be able to distinguish ion channels from those that are not. However, this will require additional work as the process of selecting non-ion channel sequences, as the training dataset would need to include a substantial number of curated sequences from distinct groups of proteins from many different species of eukaryotes.

## Conclusions

The present study delivers a practical and effective bioinformatic pipeline (MuSICC) for both the identification and classification of ion channels in parasitic flatworms of socioeconomic importance. MuSICC should be useful for the selection of high-priority candidates for functional genomic studies and for drug target discovery in parasitic flatworms. In addition, it might guide future investigations of the roles of ion channels in cellular processes and host-parasite interactions. Although applied to parasitic flatworms, the MuSICC pipeline should be applicable to classifying ion channels in a wide range of organisms.

## Additional files

Additional file 1:
**Table S1.** Sequence counts per ion channel family obtained from the KEGG and SwissProt databases and included in the *training* and *test datasets*. **Table S2.** Accession numbers of ion channels selected for support vector machine model training. **Table S3. **The number of sequences in the testing dataset before and after BLASTp analyses. **Table S4.** The number of identified *test data* sequences from humans and *C. elegans* within each group and divided into known ion channel and non-ion channel datasets. **Table S5**. Cross-validation, training and testing accuracies of each model. **Table S6.** Final tables of confusion matrices for the “Classifier” and “Dipeptide” models. **Table S7**. Summary of flatworm ion channels predicted using the MuSICC identification and classification pipeline with high and medium confidence. **Table S8**. Complete set of flatworm ion channels predicted using the MuSICC identification and classification pipeline. (XLS 2960 kb) Additional file 2:
**Figure S1.** The number of conserved domains common to >75 % of the sequences in each ion channel subfamily within the *training dataset*. The proportion of the sequences in the subfamilies that share the number of domains is given in the graph. The number of conserved domains is grouped according to the ion channel families. **Figure S2**. The range of transmembrane domains predicted in *training dataset* ion channel proteins. Range of transmembrane domains per subfamily, grouped according to each ion channel family. **Figure S3.** Receiver operating characteristic (ROC) curves for each probabilistic classification method. **Figure S4**. Probability values of each ion channel subfamilies computed during classification of sequences in the *test dataset*. Figure A shows the relation between the probability values and classifications made by SVM classifier. Figure B shows average probability values for individual ion channel subfamilies. The average values were grouped according to the ion channel families. **Figure S5**. Characteristics of putative ion channels identified and classified from the test dataset (human and *C. elegans* proteins). Panel A: Test sequences ordered by their SVM probability value, with their identification grouping presented on the second y-axis. Most of the sequences classified using high probability values were classified in *Groups 1* and *2*. Panel B: Confidence in *test data* ion channel classifications by group and classification category. (DOCX 1083 kb)
