# Supplementary material for: Pipeline for the identification and classification of ion channels in parasitic flatworms
Source: Parasit Vectors. 2016 Mar 16;9:155. doi: 10.1186/s13071-016-1428-2 (PMC4794918; doi:10.1186/s13071-016-1428-2)
Supplement: Additional file 2: — Figure S1. The number of conserved domains common to >75 % of the sequences in each ion channel subfamily within the training dataset. The proportion of the sequences in the subfamilies that share the number of domains is given in the graph. The number of conserved domains is grouped according to the ion channel families. Figure S2. The range of transmembrane domains predicted in training dataset ion channel proteins. Range of transmembrane domains per subfamily, grouped according to each ion channel family. Figure S3. Receiver operating characteristic (ROC) curves for each probabilistic classification method. Figure S4. Probability values of each ion channel subfamilies computed during classification of sequences in the test dataset. Figure A shows the relation between the probability values and classifications made by SVM classifier. Figure B shows average probability values for individual ion channel subfamilies. The average values were grouped according to the ion channel families. Figure S5. Characteristics of putative ion channels identified and classified from the test dataset (human and C. elegans proteins). Panel A: Test sequences ordered by their SVM probability value, with their identification grouping presented on the second y-axis. Most of the sequences classified using high probability values were classified in Groups 1 and 2. Panel B: Confidence in test data ion channel classifications by group and classification category. (DOCX 1083 kb) [file 13071_2016_1428_MOESM2_ESM.docx]

Parasites and Vectors

Additional file 2: Supplementary Figures

**Pipeline for the prediction and classification of ion channels in parasitic flatworms**

**Bahiyah Nor^1,a^, Neil D. Young^1,a,*^, Pasi K. Korhonen^1^, Ross Hall^1^, Patrick Tan^2,3^, Andrew Lonie^4^ and Robin B. Gasser^1,*^**

^1^Faculty of Veterinary and Agricultural Sciences, The University of Melbourne, Parkville, Victoria 3010, Australia. ^2^Genome Institute of Singapore, 60 Biopolis Street, Singapore 138672, Republic of Singapore. ^3^Cancer and Stem Cell Biology, Duke-NUS Graduate Medical School, Singapore 138672, Republic of Singapore. ^4^Victorian Life Sciences Computation Initiative (VLSCI), The University of Melbourne, Parkville, Victoria 3010, Australia.


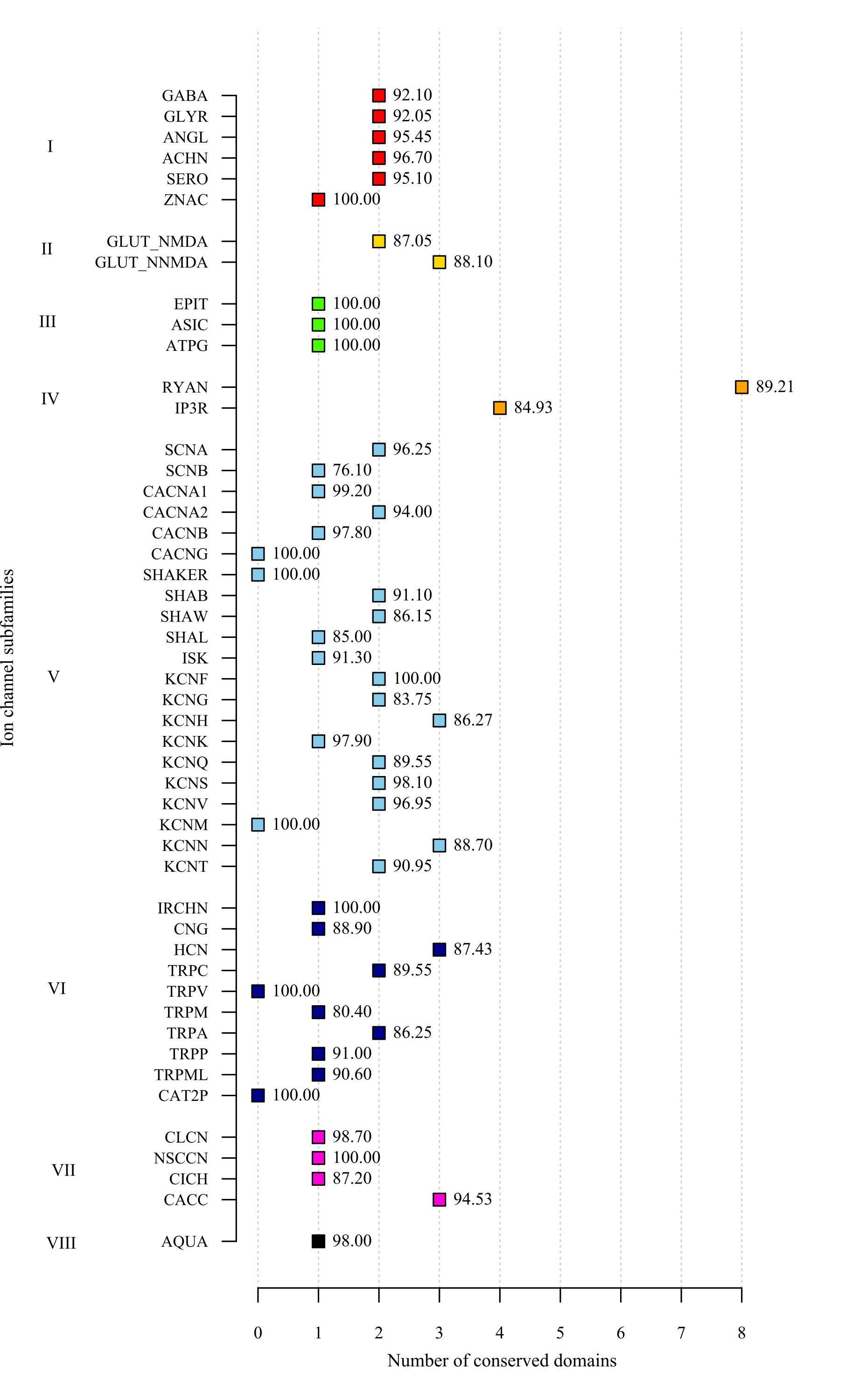


Figure S1. The number of conserved domains common in > 75% of the sequences in each ion channel subfamily within the *training dataset*. The proportion of the sequences in the subfamilies that share the number of domains is given in the graph. . The number of conserved domains is grouped according to the ion channel families. The families are: I. Cys-loop superfamily, II. Glutamate-gated cation channel, III. Epithelial and Related Channels, IV. Ryanodine and IP_3_ receptors, V. Voltage-gated cation channels, VI. Related to voltage-gated cation channels, VII. Chloride channels, and VIII. Aquaporins. The description on the labels used to represent the ion channel subfamilies is given in Supplementary Table 8.

**
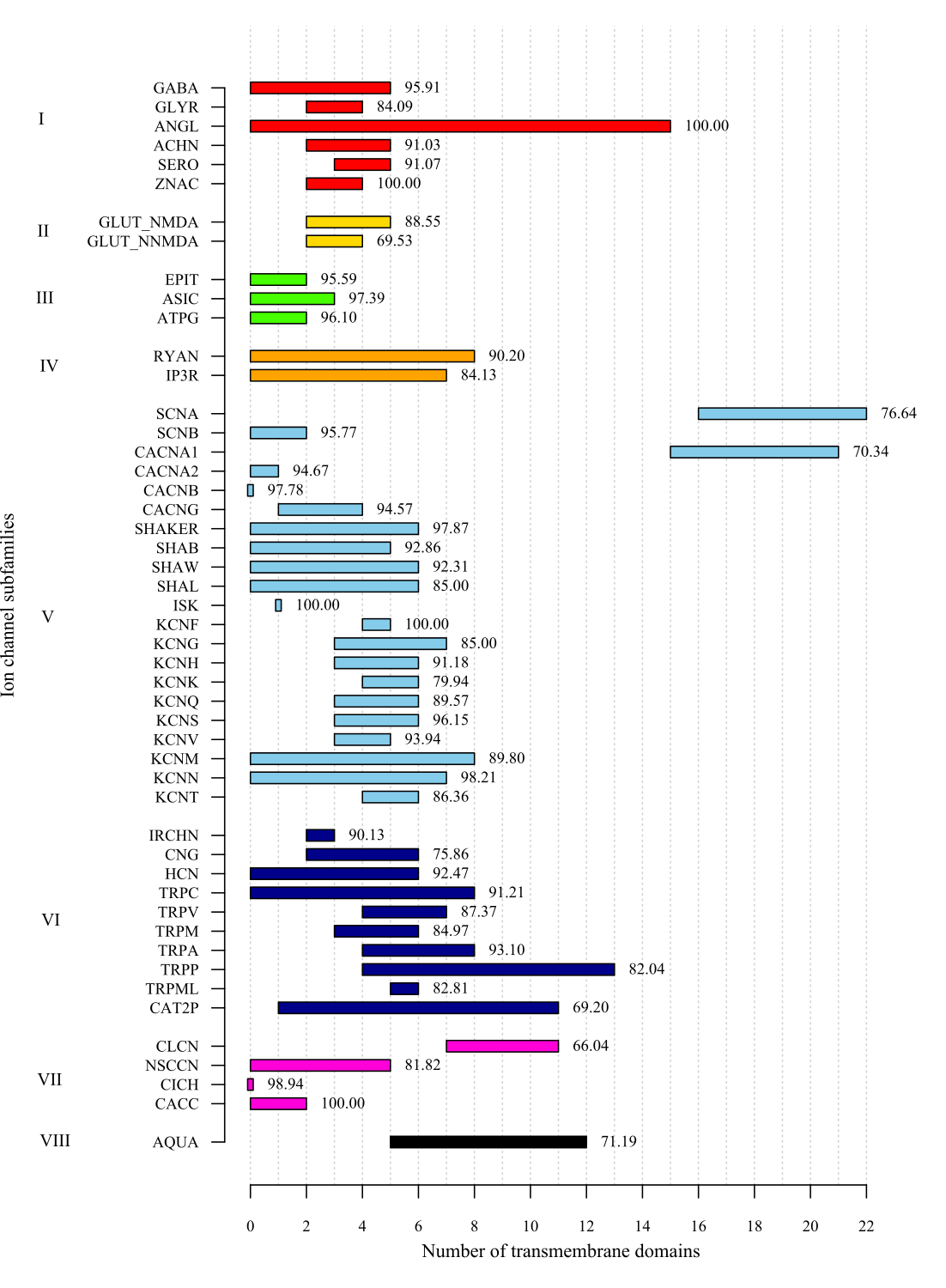
Figure S2.** The range of transmembrane domains predicted in *training dataset* ion channel proteins. Range of transmembrane domains per subfamily grouped according to each ion channel family. The families are: I. Cys-loop superfamily, II. Glutamate-gated cation channel, III. Epithelial and Related Channels, IV. Ryanodine and IP_3_ receptors, V. Voltage-gated cation channels, VI. Related to voltage-gated cation channels, VII. Chloride channels, and VIII. Aquaporins. The description on the labels used to represent the ion channel subfamilies is given in Supplementary Table 8.

**
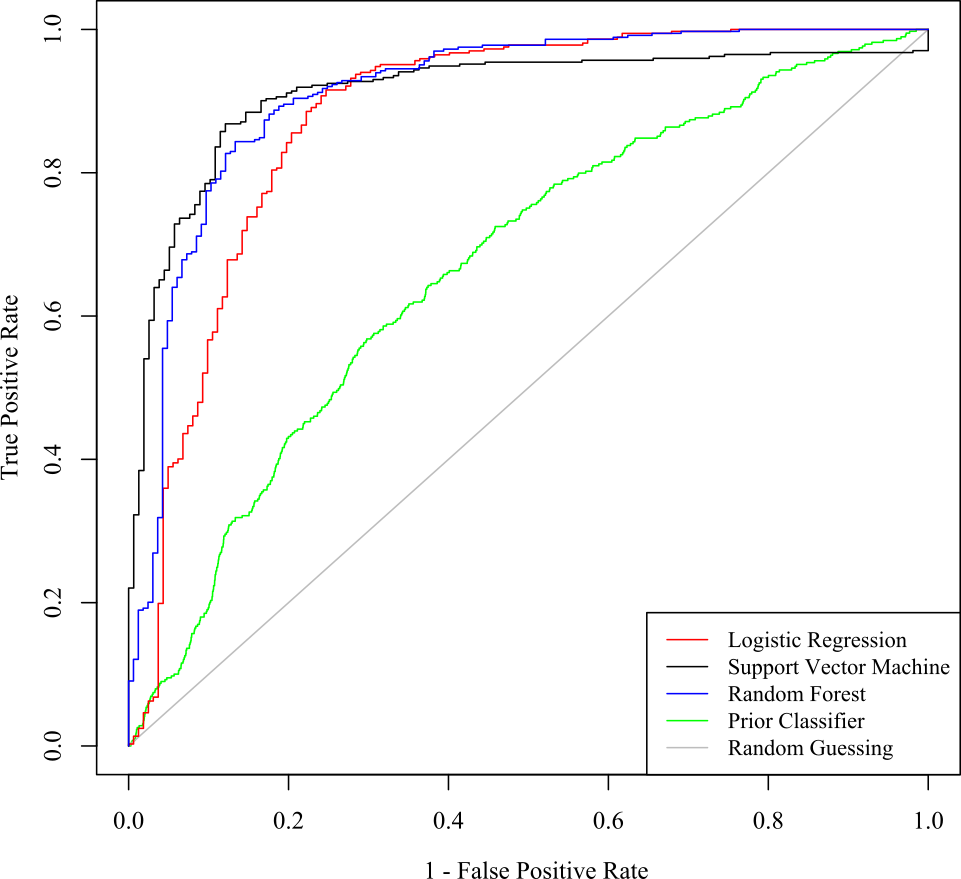
Figure S3.** Receiver operating characteristic (ROC) curves for each probabilistic classification method. The area-under-the-curve (AUC) for the support vector machine (SVM) classifier was 0.911; random forest = 0.9105; logistic regression classifier = 0.8211; and Prior classifier = 0.6701.


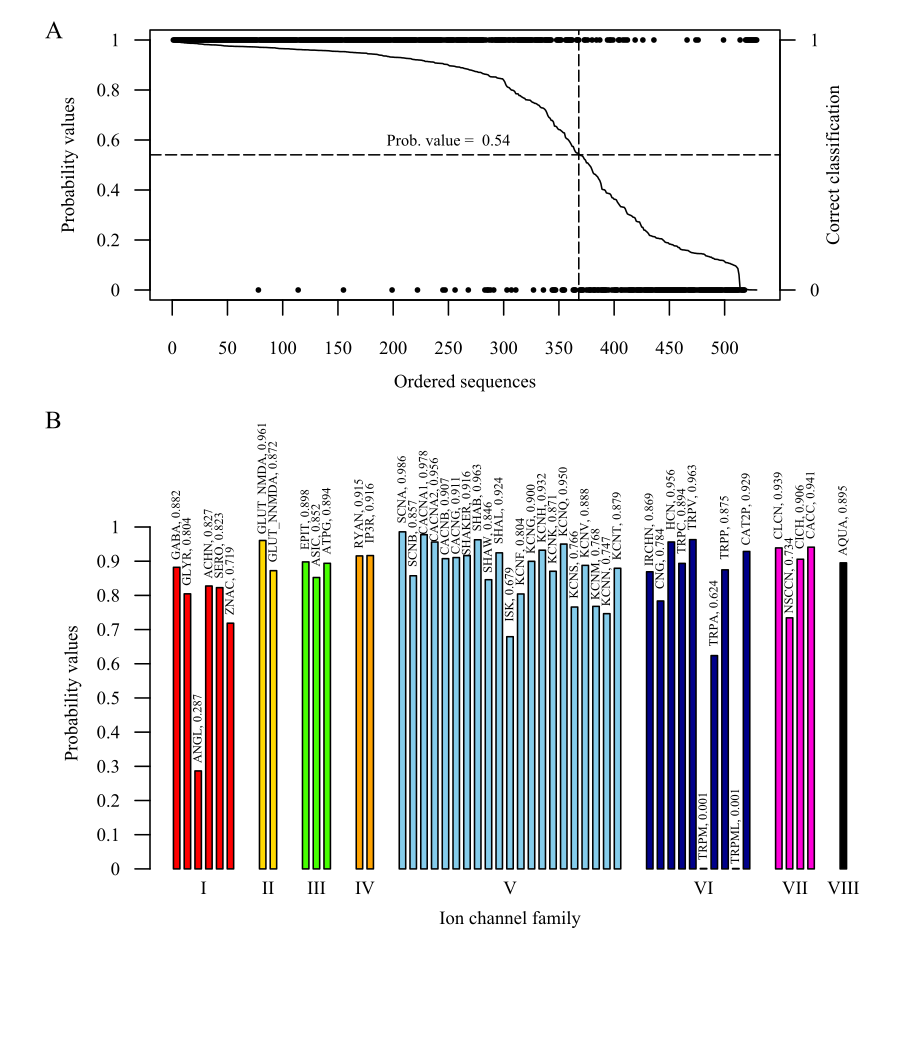


Figure S4. Probability values of each ion channel subfamilies computed during classification of sequences in the *test dataset*. Figure A shows the relation between the probability values and classifications made by SVM classifier. It was observed that as the probability values decrease, more misclassifications were made. At approximately probability equals to 0.54, a series of misclassifications were made. Figure B shows average probability values for individual ion channel subfamilies. The average values were grouped according to the ion channel families. The families are: I. Cys-loop superfamily, II. Glutamate-gated cation channel, III. Epithelial and Related Channels, IV. Ryanodine and IP_3_ receptors, V. Voltage-gated cation channels, VI. Related to voltage-gated cation channels, VII. Chloride channels, and VIII. Aquaporins. The average probability values vary between the families and the subfamilies. The description on the labels used to represent the ion channel subfamilies is given in Supplementary Table 8.

**
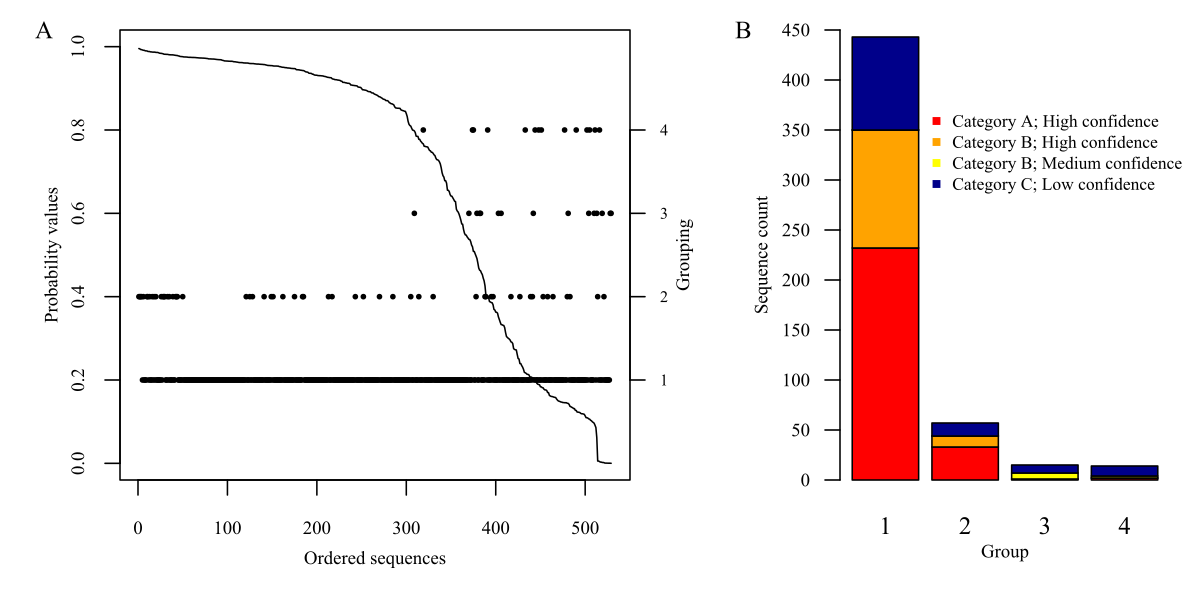
Figure S5.** Characteristics of putative ion channels identified and classified from the test dataset (human and *C. elegans* proteins). Panel A: Test sequences ordered by their SVM probability value with their identification grouping presented on the second y-axis. Most of the sequences classified using high probability values were classified in Groups 1 and 2. Panel B: Confidence in *test data* ion channel classifications by group and classification category.
